# Association between duration of electronic screen use for non-educational purposes and depression symptoms among middle and high school students: a cross-sectional study in Zhejiang Province, China

**DOI:** 10.3389/fpubh.2023.1138152

**Published:** 2023-05-16

**Authors:** Hao Wang, Fiona Bragg, Yunqi Guan, Jieming Zhong, Na Li, Jin Pan, Min Yu

**Affiliations:** ^1^Department of NCDs Control and Prevention, Zhejiang Provincial Center for Disease Control and Prevention, Hangzhou, China; ^2^Medical Research Council Population Health Research Unit, Nuffield Department of Population Health, University of Oxford, Oxford, United Kingdom; ^3^Clinical Trial Service Unit and Epidemiological Studies Unit (CTSU), Nuffield Department of Population Health, University of Oxford, Oxford, United Kingdom

**Keywords:** depression symptoms, electronic screen, adolescents, cross-sectional study, factors

## Abstract

**Background:**

Existing literature on the association of electronic screen use duration with depression among adolescents is contradictory. The current study aimed to elucidate the association between duration of electronic screen use for non-educational purposes and depression symptoms among middle and high school students in Zhejiang Province, China.

**Methods:**

A cross-sectional study of 27,070 students in grades 7–12 from 376 middle and high schools was conducted through an anonymous self-administered questionnaire between April and June 2022. Poisson regression was utilized to examine the association between electronic screen use duration for non-educational purposes and depression symptoms.

**Results:**

Of the 27,006 eligible students, 51.6% (13932) were boys and the mean (SD) age was 15.6(1.7) years. The overall prevalence of symptoms of depression was 22.4% (95%CI 21.4–23.4); girls (27.6%, 26.2–29.0) had a higher prevalence than boys (17.7%, 16.7–18.8). After adjustment for socio-demographic status, lifestyle factors, self-perceived health, academic performance, loneliness and sadness, compared to those who did not use electronic screens for non-educational purposes, the prevalence ratios (PRs) for depression symptoms were 1.03 (95% CI 1.02–1.04) for those exposed to electronic screens for <1 h/day, 1.07 (1.05–1.09) for 1.0–1.9 h/day, 1.10 (1.07–1.13) for 2.0–2.9 h/day, 1.14 (1.10–1.18) for 3.0–3.9 h/day, 1.18 (1.12–1.23) for 4.0–4.9 h/day, and 1.21 (1.15–1.29) for ≥5 h/day.

**Conclusion:**

Duration of electronic screen use for non-educational purposes was positively associated with symptoms of depression among middle and high school students, even with a relatively short daily duration of use.

## Introduction

Mental disorders are increasingly recognized as leading causes of disease burden (1). The estimated number of mental disorders worldwide increased from 654.8 million in 1990 to 970.1 million in 2019, corresponding to an increase of 48.1% (2). Depression is the most common type of mental disorder (3), and was estimated to have increased worldwide from 170.8 million to 279.6 million between 1990 and 2019 (2). Globally, 8.8% of children and adolescents (<20 years old) were diagnosed with mental disorders in 2019 (1). Among this age group, the frequency of depression increased from 819.5 per 100,000 in 2010 to 908.6 per 100,000 in 2019 (1), and disability adjusted life years (DALYs) due to depression increased from 3.5 million to 4.4 million in the same period.

With rapid development of economies and technology, the variety of electronic devices available has increased dramatically over recent decades, to include not only traditional televisions and desktop computers, but also smart phones, laptops, and tablet devices etc. In the United States of America, the percentage of high school students using electronic screens for longer than 3 h per day increased from 22.1% in 2003 to 46.1% in 2019 (4). These electronic devices greatly facilitate people’s lives, but potential adverse effects on users’ health have led to concerns regarding excessive exposure to electronic screens. Previous studies documented that excessive screen time was associated with a wide range of detrimental health behaviors among adolescents, including insufficient sleep (5, 6), excessive sugar-sweetened beverage consumption (6) and inadequate physical activity (6). In addition, excessive screen time has also been associated with obesity (6, 7), metabolic syndrome (8), insulin resistance (9), attention deficit and hyperactivity disorder (10) and anxiety symptoms (11).

However, existing literature on the association of duration of electronic screen use with depression among adolescents is contradictory. While some studies indicated that there was a significant positive association between screen time and depression (12–14), one study conducted in Taiwan found that time spent playing Internet games appeared to be negatively associated with depression symptoms (15), and similar results were documented by another nationally representative study of 9,137 Canadian adolescents aged 12–19 years, which indicated that depression was less likely to be reported in frequent video game users (16). The School Children Mental Health Europe project, including 3,195 children aged 6–11 years from six European Union countries, found no significant association between video game playing and depression (17). Hence, the current study was designed with the aim of evaluating the association between duration of viewing electronic screens for non-educational purposes and depression symptoms among school students in Zhejiang.

## Materials and methods

### Study design

A three-stage cluster sampling design was implemented. In stage 1, 30 counties/districts were sampled randomly from all 90 counties/districts. In stage 2, 11 classes of middle school, 6 classes of academic high school, and 6 classes of vocational high school were selected randomly within each chosen county/district. In stage 3, students in all selected classes were invited to participate in the study. A self-administered anonymous questionnaire was filled in by participants in the classroom setting without school teachers’ supervision. The field survey was carried out by trained CDC staff using standardized procedures.

### Outcome variables

Depressive symptoms were assessed using the Patient Health Questionnaire 9-item depression scale (PHQ-9) (18), widely used among adolescents (19–22). Participants were asked to report the presence of nine problems, including depression and interest decline, in the past 2 weeks on a 4-point scale ranging from “nearly every day” (3 points) to “not at all” (0 points). The response to each item can be assigned a score of 0 to 3, with a maximum combined score of 27. Higher scores indicate greater severity of depressive symptoms. Previous studies recommended a cut-off point of no less than 10 to screen for depression of clinical diagnostic significance of at least moderate severity (18). Levis et al. demonstrated high specificity (85%) and high sensitivity (88%) of the PHQ-9 scale in detecting depression at a cut-off of 10 (23). Hence, depression was defined based on a cut-off point of no less than 10 in the current study, and moderate or more severe depression was defined using a cut-off point of no less than 15 (24).

### Exposure variables

Duration of electronic screen use for non-educational purposes was evaluated through the question: “On an average school day, how many hours do you play pad or use a smartphone or tablet computer or desktop computer for something that is not school work?” Response options included: “I do not play pad or use a smartphone or tablet computer or desktop computer or play these only for school work,” “<1 h/day,” “1.0–1.9 h/day,” “2.0–2.9 h/day,” “3.0–3.9 h/day,” “4.0–4.9 h/day,” and “≥5 h/day.”

### Covariates

Covariates in the present study included age, sex, region, type of school, parental educational attainment, parental marital status, household income, cigarette smoking (smoking on at least 1 day in the past 30 days), alcohol drinking (drinking alcohol on at least 1 day in the past 30 days), physical activity, academic performance, self-perceived health, loneliness and sadness. Sadness was assessed through the question: “During the past 12 months, did you ever feel so sad or hopeless almost every day for 2 weeks or more in a row that you stopped doing some usual activities?” (Answer options: yes and no).

### Statistical analysis

Continuous variables were presented as mean ± standard deviation. Categorical variables were presented as percent and 95% confidence intervals (CI). Weighted prevalence was calculated using the PROC SURVEYFREQ procedure. Considering high prevalence of depression symptoms, modified Poisson regression was utilized to examine the associations between duration of electronic screen use for non-educational purposes and depression symptoms (25). Potential confounding factors, comprising socio-demographic status, lifestyle factors, academic performance, self-perceived health, loneliness and sadness were included in different models. Prevalence ratios (PR_S_) were estimated using three regression models. In model 1, PR_S_ were adjusted for age (≤13, 14–15, and ≥16 years), sex (boys and girls), region (urban and rural), and type of school (middle school, academic high school and vocational high school). Model 2, additionally adjusted for paternal and maternal educational attainment (middle school or below, high school, college or above) and parental marital status (married and others), household income (very poor/poor, fair, very wealthy/wealthy), cigarette smoking (yes and no), alcohol drinking (yes and no), physical activity (none, 1–2 days/week, 3–5 days/week, and 6–7 days/week), academic performance (excellent, middle and poor), and self-perceived health status (very good/good, fair, very bad/bad, and unknown). Model 3, additionally adjusted for loneliness (never/occasional, sometimes, and often/always) and sadness (yes and no). In multiple linear regression analyses, the exposure variable (i.e., duration of exposure to electronic screens for non-educational purposes) was converted to a continuous variable. Those who chose “<1 h/day,” “1.0–1.9 h/day,” “2.0–2.9 h/day,” “3.0–3.9 h/day,” “4.0–4.9 h/day,” and “≥5 h/day” were assigned with value 0.5 h/day, 1.5 h/day, 2.5 h/day, 3.5 h/day, 4.5 h/day, and 5.5 h/day, respectively. The association between duration of electronic screen use for non-educational purposes and depression symptoms was further examined. In sensitivity analyses, a cut-point of 15 was used to define depression for evaluation of its association with duration of screen use. All statistical analyses were performed using SAS software V.9.4 (SAS Institute, Cary, North Carolina, United States). Statistical significance level was set at *p* value < 0.05 using a 2-sided test.

## Results

### Descriptive statistics

A total of 28,043 students from 376 schools were invited to participate, of whom, 27,070 students were surveyed. Overall, 114 students refused to participate and 859 were absent from school on the survey day, yielding a response rate of 96.5%. Of 27,070 questionnaires, 40 were excluded due to being incomplete, 17 were excluded due to missing one of more of the nine items in the PHQ-9 questionnaire, and 7 were excluded due to missing information on duration of electronic screen use. Eventually, 27,006 students, comprising 13,932 boys and 13,074 girls, were included in the present analyses. The mean age was 15.6 ± 1.7 years. The percentages of students attending middle schools, academic high schools and vocational high schools were 47.2% (*N* = 12,760), 27.3% (*N* = 7,375), and 25.5% (*N* = 6,871), respectively.

Of 27,006 eligible students, 35.3% of students lived in urban areas, 87.6% grew up in intact families, and 5.5% described their family income as very poor or poor. The fathers of 55.2% of students were educated to middle school level or below, and the mothers of 58.7% of students were educated to that level. Overall, 30.5% of students described their academic performance as poor, 3.9% smoked cigarettes, and 16.0% drank alcohol. 17.3% were physically active no less than 6 days weekly. Poor health was self-reported by 7.6% of students, 13.1% felt lonely often or always in the past 12 months and 17.8% felt sad in the past 12 months (Table 1).

**Table 1:** Participant characteristics by duration of exposure to electronic screens for non-educational purposes (*N* = 27,006).

	Daily duration of exposure to electronic screens for non-educational purposes						
Characteristics	None	<1 h	1.0–1.9 h	2.0–2.9 h	3.0–3.9 h	4.0–4.9 h	≥5 h
	(*N* = 9,056)	(*N* = 5,100)	(*N* = 3,079)	(*N* = 2,759)	(*N* = 2,311)	(*N* = 1,580)	(*N* = 3,121)
Mean age (years)	15.6 ± 1.7	15.1 ± 1.7	15.4 ± 1.8	15.7 ± 1.7	16.1 ± 1.6	16.3 ± 1.5	16.3 ± 1.5
Boys (%)	4,754 (53.5)	2,572 (50.8)	1,516 (50.3)	1,403 (52.3)	1,178 (52.1)	796 (51.2)	1713 (56.1)
Urban (%)	3,538 (35.1)	2,337 (41.5)	1,332 (37.5)	1,140 (35.2)	917 (35.3)	609 (33.5)	917 (24.9)
Middle school (%)	4,400 (52.1)	3,243 (68.5)	1730 (62.2)	1,286 (52.4)	807 (39.9)	459 (33.9)	835 (30.6)
Living in intact families (%)	8,089 (89.9)	4,523 (89.0)	2,704 (88.3)	2,378 (86.4)	1972 (86.3)	1,323 (84.5)	2,530 (81.2)
Father educated to middle school level or below (%)	4,513 (49.1)	2,586 (50.6)	1715 (55.6)	1,621 (58.7)	1,405 (61.3)	1,024 (65.2)	2077 (68.3)
Mother educated to middle school level or below (%)	4,918 (53.3)	2,720 (52.8)	1814 (58.1)	1712 (62.7)	1,535 (66.4)	1,065 (66.9)	2,213 (71.5)
Low family income (%)	468 (4.8)	268 (4.9)	154 (4.9)	143 (5.0)	143 (5.8)	116 (7.1)	281 (8.8)
Physically active ≥6 d/wk. (%)	1,699 (18.8)	1,005 (19.2)	503 (16.4)	483 (17.8)	349 (14.6)	215 (13.6)	433 (14.3)
Poor academic performance (%)	2,232 (24.4)	1,434 (27.4)	912 (30.2)	960 (35.0)	830 (36.6)	606 (38.8)	1,272 (40.8)
Cigarette smoking (%)	187 (1.9)	120 (2.2)	66 (2.0)	133 (4.8)	106 (4.5)	121 (7.5)	379 (11.4)
Alcohol drinking (%)	1,099 (12.0)	627 (11.6)	406 (13.2)	489 (17.4)	498 (20.4)	382 (24.1)	959 (29.5)
Poor self-reported health (%)	635 (7.0)	347 (6.9)	204 (6.7)	197 (7.1)	154 (6.5)	123 (7.9)	361 (12.0)
Feeling lonely often or always (%)	1,056 (12.4)	635 (12.8)	331 (11.0)	301 (11.3)	295 (13.1)	217 (13.8)	588 (19.3)
Feeling sad (%)	1,331 (14.8)	850 (16.5)	515 (16.9)	499 (18.8)	447 (19.7)	313 (20.0)	823 (26.2)

Number in brackets are weighted proportions.

### Prevalence of depression symptoms

The overall prevalence (95%CI) of depression symptoms among students was 22.4% (21.4–23.4), and was 20.1% (18.4–21.9), 23.6% (21.9–25.2), and 22.7% (21.3–24.2) for students aged ≤13 years, 14–15 years, and ≥16 years, respectively (*p* = 0.01). Girls (27.6, 95%CI: 26.2–29.0) had a higher prevalence of depression symptoms than boys (17.7, 95%CI:16.7–18.8). Students living in rural areas (23.8, 95%CI: 22.5–25.2) had a higher prevalence of depression symptoms than their counterparts living in urban areas (19.9, 95%CI: 18.7–21.0). Prevalence of depression symptoms among students attending middle school, academic high school, and vocational high school was 22.1% (95%CI: 20.7–23.5), 24.6% (95%CI: 22.4–26.7), and 20.8% (95%CI: 18.9–22.6), respectively (*p* = 0.03; Table 2).

**Table 2:** Weighted prevalence of depression symptoms by participant characteristics.

	Prevalence (95%CI)*	*p* value
Age (years)		0.01
≤13	20.1 (18.4–21.9)	
14–15	23.6 (21.9–25.2)	
≥16	22.7 (21.3–24.2)	
Sex		<0.001
Boys	17.7 (16.7–18.8)	
Girls	27.6 (26.2–29.0)	
Area		<0.001
Urban	19.9 (18.7–21.0)	
Rural	23.8 (22.5–25.2)	
Type of school		0.03
Middle school	22.1 (20.7–23.5)	
Academic high school	24.6 (22.4–26.7)	
Vocational high school	20.8 (18.9–22.6)	

* Based on the weighted data. CI, confidence interval.

### Association between duration of electronic screen use for non-educational purposes and depression symptoms

After adjusting for age, sex, region, type of school, paternal and maternal educational attainment, parental marital status, household income, cigarette smoking, alcohol drinking, physical activity, academic performance, self-perceived health, loneliness and sadness, compared to those who were not exposed to electronic screens, the PRs (95%CI) for depression symptoms were 1.03 (1.02–1.04) for those exposed to electronic screens for <1 h/day, 1.07 (1.05–1.09) for those exposed for 1.0–1.9 h/day, 1.10 (1.07–1.13) for those exposed for 2.0–2.9 h/day, 1.14 (1.10–1.18) for those exposed for 3.0–3.9 h/day, 1.18 (1.12–1.23) for those exposed for 4.0–4.9 h/day, and 1.21 (1.15–1.29) for those exposed for ≥5 h/day, respectively (Table 3). There was an apparent exposure-response effect such that those exposed to electronic screens for a longer duration had a greater prevalence of depression symptoms. In multiple linear regression analyses, the adjusted β coefficients (95%CI) of association between duration of electronic screen use for non-educational purposes and depression symptoms scores was 0.14 (0.10–0.17), and the same pattern was also found among boys and girls (all *p* values < 0.001; Table 4).

**Table 3:** Adjusted prevalence ratios of depression symptoms associated with duration of exposure to electronic screens for non-educational purposes among students.

	Daily duration of exposure to electronic screens for non-educational purposes							*p* for trend
	None (*N* = 9,056)	<1 h (*N* = 5,100)	1.0–1.9 h (*N* = 3,079)	2.0–2.9 h (*N* = 2,759)	3.0–3.9 h (*N* = 2,311)	4.0–4.9 h (*N* = 1,580)	≥5.0 h (*N* = 3,121)	
Total								
Model 1	1.00	1.10 (1.09–1.11)	1.21 (1.18–1.24)	1.33 (1.29–1.38)	1.46 (1.40–1.53)	1.61 (1.52–1.70)	1.77 (1.66–1.89)	<0.001
Model 2	1.00	1.05 (1.04–1.07)	1.11 (1.09–1.14)	1.17 (1.14–1.21)	1.24 (1.18–1.29)	1.30 (1.24–1.38)	1.38 (1.29–1.47)	<0.001
Model 3	1.00	1.03 (1.02–1.04)	1.07 (1.05–1.09)	1.10 (1.07–1.13)	1.14 (1.10–1.18)	1.18 (1.12–1.23)	1.21 (1.15–1.29)	<0.001
Boys								
Model 1	1.00	1.10 (1.08–1.12)	1.21 (1.17–1.25)	1.33 (1.26–1.40)	1.47 (1.37–1.57)	1.61 (1.48–1.76)	1.77 (1.60–1.97)	<0.001
Model 2	1.00	1.05 (1.04–1.07)	1.11 (1.07–1.15)	1.17 (1.11–1.23)	1.23 (1.15–1.32)	1.30 (1.19–1.42)	1.37 (1.23–1.52)	<0.001
Model 3	1.00	1.04 (1.02–1.05)	1.08 (1.04–1.11)	1.12 (1.07–1.17)	1.16 (1.09–1.23)	1.20 (1.12–1.30)	1.25 (1.14–1.37)	<0.001
Girls								
Model 1	1.00	1.10 (1.08–1.11)	1.21 (1.18–1.24)	1.33 (1.27–1.39)	1.46 (1.38–1.54)	1.61 (1.50–1.72)	1.77 (1.62–1.92)	<0.001
Model 2	1.00	1.05 (1.04–1.07)	1.11 (1.08–1.14)	1.17 (1.12–1.22)	1.24 (1.17–1.31)	1.30 (1.22–1.39)	1.37 (1.26–1.49)	<0.001
Model 3	1.00	1.03 (1.02–1.04)	1.06 (1.03–1.09)	1.09 (1.05–1.13)	1.12 (1.07–1.18)	1.16 (1.09–1.23)	1.19 (1.11–1.28)	<0.001

Model 1, prevalence ratios were adjusted for age, sex, region, and type of school. Model 2, additionally adjusted for paternal and maternal educational attainment, parental marital status, family income, cigarette smoking, alcohol drinking, physical activity, academic performance, and self-perceived health. Model 3, additionally adjusted for loneliness and sadness.

**Table 4:** Adjusted β coefficients for scores of depression symptoms associated with duration of exposure to electronic screens for non-educational purposes among students.

	Adjusted β (95%CI)	*p* value
Total		
Model 1	0.36 (0.31–0.41)	<0.001
Model 2	0.21 (0.17–0.25)	<0.001
Model 3	0.14 (0.10–0.17)	<0.001
Boys		
Model 1	0.32 (0.27–0.37)	<0.001
Model 2	0.19 (0.14–0.24)	<0.001
Model 3	0.14 (0.09–0.19)	<0.001
Girls		
Model 1	0.41 (0.34–0.48)	<0.001
Model 2	0.23 (0.17–0.29)	<0.001
Model 3	0.13 (0.08–0.17)	<0.001

β coefficients (95%CI) represents changes in score of depression symptoms per hours/day difference in duration of electronic screen. Model 1, β coefficients were adjusted for age, sex, region, and type of school. Model 2, additionally adjusted for paternal and maternal educational attainment, parental marital status, family income, cigarette smoking, alcohol drinking, physical activity, academic performance, and self-perceived health. Model 3, additionally adjusted for loneliness and sadness.

### Sensitivity analyses

In sensitivity analyses, a cut-off value of 15 (i.e., moderately or more severe depression) was used to evaluate the association of duration of electronic screens viewing with depression symptoms. After adjusting for potential confounding factors, compared to those who were not exposed to electronic screens, the PR (95%CI) for depression symptoms was 1.04 (1.03–1.06) for those exposed to electronic screens for non-educational purposes for <1 h/day, 1.09 (1.05–1.13) for those exposed for 1.0–1.9 h/day, 1.14 (1.08–1.20) for those exposed for 2.0–2.9 h/day, 1.19 (1.11–1.27) for those exposed for 3.0–3.9 h/day, 1.24 (1.14–1.35) for those exposed for 4.0–4.9 h/day, and 1.30 (1.17–1.44) for those exposed for ≥5 h/day, respectively, which was similar to the results based on a cut-off value of 10 (Additional file 1: Supplementary Table S1).

## Discussion

This provincially representative study of middle and high school students from China documents the latest prevalence of depression symptoms, and quantifies the associations between duration of electronic screen use for non-educational purposes and depression symptoms.

### Prevalence of depression symptoms

A meta-analysis of 29 studies including 80,879 global youth aged ≤18 years indicated that the prevalence of depression symptoms was 25.2% (26). The prevalence of depression symptoms in the current study was 22.4%, suggesting that these symptoms are common among school students in China. In 2021, the Chinese government took a series of actions to strengthen the prevention of adolescent depression, including adding a depression screen to routine student health checks, establishing student mental health archives and student awareness of depression, and strengthening capacity for early recognition of depression (27). Consistent with previous studies, the prevalence of depression symptoms among girls was higher than among boys (28–30), implying that girls were more inclined to suffer from depression. In line with a previous study (29), the prevalence of depression symptoms increased with age. This may reflect older students encountering more triggers of depression owing to their greater autonomy and social adjustment.

### Association between duration of electronic screen use for non-educational purposes and depression symptoms

Consistent with the majority of previous studies (31–33), electronic screen use was positively associated with depression symptoms in the current study. The 2018 US National Survey of Children’s Health (NSCH), including 10,907 adolescents aged 13 to 17 years, observed that after adjusting for demographic factors (age, sex, poverty level, parent education, and race/ethnicity), insurance type, language spoken at home, household generation, family structure, comorbid conditions, and emotional/behavior medications, compared to adolescents with electronic screen duration of less than 1 h/day, the odd ratios (95%CI) of depression for those exposed to electronic screens for 1 h/day, 2 h/day, 3 h/day, and ≥ 4 h/day were 0.66 (0.34–1.28), 1.26 (0.71–2.25), 1.48 (0.83–2.63), and 2.23 (1.27–3.91), respectively; only those children using screens for ≥4 h/day had significantly higher odds of depression (34). The less clear association between duration of screen use in this US study, when compared with the current study, may reflect multiple factors. Firstly, information on screen time and depression was provided by students’ parents or caregivers in the NSCH, while information was provided by students themselves in the present study. Secondly, depression was assessed through history of physician-diagnosed depression in NSCH, whereas depression was identified with a self-administered PHQ-9 questionnaire in the present study.

Interestingly, the 2002 Swiss Multicentre Adolescent Survey on Health, including 7,211 students aged 16 to 20 years, found a U-shaped relationship between intensity of Internet use and depression scores. When compared with regular users (i.e., those using the Internet several days per week and ≤ 2 h/day), the risk of depression was higher among non-users (i.e., no use in the past month), occasional users (i.e., ≤1 h/week), and heavy users (>2 h/day) (12). Another study of 221,096 adolescents, included in three large datasets from the UK and USA, indicated that light users of digital media (<1 h/day) reported more favorable psychological well-being (based on suicidal thoughts and attempt, depression, etc.) than heavy users (≥5 h/day), and non-users of digital media experienced poorer psychological well-being than light users (35). The authors concluded that light users, rather than non-and moderate users, had the most favorable psychological well-being (35). In contrast with these two previous studies, which suggested that mild or moderate exposure to electronic screens was beneficial with regards to risk of depression among adolescents, a linear exposure-response relationship of duration of electronic screen use and depression symptoms was documented in the present study.

Although the underlying processes through which screen time could be related to the development of depression are likely to be complicated, several possible hypotheses have been proposed. First, excessive screen time has been associated with unhealthy behaviors, including inadequate physical activity and poor sleep quality, which may exacerbate depression symptoms (36, 37). Second, adolescents using screens for a greater duration of time are inclined to self-isolate, which might increase the likelihood of depression (38). Third, increased screen time may expose adolescents to cyberbullying, which may cause depression (39, 40).

The findings of the present study are of practical public health importance, and provide crucial evidence that may inform prevention of adolescent depression. First, more than one fifth of the students suffered from depression symptoms, implying that depression is common among students of this age, and more comprehensive efforts are needed to address adolescent mental health issues in China. These might include each middle and high school being equipped with at least one full-time mental health teacher, incorporating mental health education into the school curriculum, cultivating students’ capability of solving mental crisis, strengthening early screening for depression, and providing professional medical services for those with severe depression (27). Second, adolescents may benefit from electronic devices. For example, through providing adolescents with new ideas, information and learning opportunities (41). However, an inverse association was observed in the current study between duration of screen use and depression symptoms, and it is noteworthy that adolescents exposed to electronic screens for <1 h/day still had higher odds of depression symptoms than those without any screen use. The findings suggest that there is no “safe threshold” for the duration of electronic screen use. How to balance the benefits and adverse outcomes that may result from viewing electronic screens is a vital public health issue. Furthermore, the conclusions of the current study need to be further validated through large longitudinal studies. In 2013, the American Academy of Pediatrics (AAP) recommended screen time of less than 2 h per day for children ≥2 year of age and discouraged screen exposure for children <2 year of age (42). In 2016, AAP released an update and recommended screen time of less than 1 h per day for children aged 2–5 years, and “individualized screen time” (i.e., did not have specific recommendations on time spent on electronic screen use) for children and adolescents aged 6–18 years (41). 2021 Physical Activity Guidelines in China recommended screen time of less than 1 h per day for children aged 3–5 years, and less than 2 h per day for children and adolescents aged 6–17 years (43). The results of the current study highlight that a more flexible and individualized screen time threshold may be more appropriate for Chinese adolescents.

There are several limitations of the present study. First, the cross-sectional study design prohibits establishment of the temporal relationship between screen use and depression symptoms. Second, we did not collect the information on the type of electronic device used, which may be important since previous studies have indicated that the associations between screen behavior and depression varied by screen behavior types (44). Third, all data came from self-report, and the findings presented may be susceptible to recall or social desirability biases. Fourth, assessment of exposure to electronic screens in the current study did not include television watching, which might underestimate real screen time.

## Conclusion

In summary, our study sheds light on the association between duration of electronic screen use for non-educational purposes and depression symptoms among middle and high school students in Zhejiang Province, China. We found that depression symptoms were prevalent, and duration of electronic screen use for non-educational purposes was positively associated with depression symptoms among students, despite short-duration of exposure. It may be appropriate to include reduced electronic screen time in targeted prevention of adolescent depression.

## Data availability statement

The raw data supporting the conclusions of this article will be made available by the authors, without undue reservation.

## Ethics statement

The studies involving human participants were reviewed and approved by Ethics Committee of Zhejiang Provincial Centre for Disease Control and Prevention. Written informed consent to participate in this study was provided by the participants’ legal guardian/next of kin.

## Author contributions

HW designed the study, drafted the manuscript, and analyzed the data. NL, YG, and JP collected the data. JZ and MY were involved in data interpretation. FB provided important comments on the manuscript and revised the manuscript. All authors contributed to the article and approved the submitted version.

## Funding

This work was supported by Program of Zhejiang Federation of Humanities and Social Sciences (grant number 2023B059).

## Conflict of interest

The authors declare that the research was conducted in the absence of any commercial or financial relationship that could be construed as a potential conflict of interest.

## Publisher’s note

All claims expressed in this article are solely those of the authors and do not necessarily represent those of their affiliated organizations, or those of the publisher, the editors and the reviewers. Any product that may be evaluated in this article, or claim that may be made by its manufacturer, is not guaranteed or endorsed by the publisher.
