# Supplementary material for: Association between duration of electronic screen use for non-educational purposes and depression symptoms among middle and high school students: a cross-sectional study in Zhejiang Province, China
Source: Front Public Health. 2023 May 16;11:1138152. doi: 10.3389/fpubh.2023.1138152 (PMC10229063; doi:10.3389/fpubh.2023.1138152)
Supplement: Supplementary file 1 [file Table_1.docx]

**Table S1 Adjusted prevalence ratios of depression symptoms with a cut-off value of 15 associated with duration of exposure to electronic screens for non-educational purposes**

|  | Daily duration of exposure to electronic screen for non-educational purposes | | | | | | | *P* for trend |
| --- | --- | --- | --- | --- | --- | --- | --- | --- |
|  | None  (N=9 056) | <1 hours  (N=5 100) | 1.0-1.9 hours  (N=3 079) | 2.0-2.9 hours  (N=2 759) | 3.0-3.9 hours  (N=2 311) | 4.0-4.9 hours  (N=1 580) | ≥5.0 hours  (N=3 121) |  |
| Total | 1.00 | 1.04 (1.03-1.06) | 1.09 (1.05-1.13) | 1.14 (1.08-1.20) | 1.19 (1.11-1.27) | 1.24 (1.14-1.35) | 1.30 (1.17-1.44) | <0.001 |
| Boys | 1.00 | 1.07 (1.04-1.10) | 1.14 (1.07-1.20) | 1.21 (1.11-1.32) | 1.29 (1.15-1.44) | 1.38 (1.20-1.58) | 1.47 (1.24-1.73) | <0.001 |
| Girls | 1.00 | 1.03 (1.01-1.05) | 1.06 (1.02-1.11) | 1.09 (1.03-1.17) | 1.13 (1.04-1.23) | 1.16 (1.04-1.29) | 1.20 (1.05-1.36) | <0.001 |

Prevalence ratios were adjusted for age, sex, type of school, paternal and maternal educational attainment, parental marital status, family income, cigarette smoking, alcohol drinking, physical activity, academic performance, self-perceived health, loneliness and sadness.
